# PREDICTING DYADIC RELATIONSHIP STRAIN IN INDIVIDUALS WITH DEMENTIA

**DOI:** 10.1093/geroni/igac059.2795

**Published:** 2022-12-20

**Authors:** Amanda MacNeil, Katherine Judge, David Bass

**Affiliations:** Cleveland State University, Fairview Park, Ohio, United States; Cleveland State University, Cleveland, Ohio, United States; The Benjamin Rose Institute on Aging, Cleveland, Ohio, United States

## Abstract

Recent literature has addressed the perceptions of individuals with dementia to understand how they experience their illness, with evidence suggesting these perceptions are impactful. Few studies, however, have used a conceptual model to explore different aspects of the illness. One aspect to consider is dyadic relationship strain, or feelings of tension, manipulation, and stress between the individual and caregiver. Little work has addressed this strain from the perspective of the individual with dementia who may have different feelings about the quality of the relationship. Cognition and function are two hallmark symptoms in dementia, however little work has addressed how the perception of these two areas impacts the illness experience. Perceptions of difficulties in these two areas may impact dyadic relationship strain as they necessitate increased care and changes in the relationship. Guided by the Stress Process Model for Individuals with Dementia, this study assessed potential predictors of dyadic relationship strain, finding personal activities of daily living (PADLs) to be impactful. In a multiple regression, PADLS (b=.319, p = 02) predicted strain above and beyond two measures of cognition: objective cognitive impairment (b =-.011, p =.93) and perceived memory difficulty (b=.003, p=.311) suggesting that perceived function is impactful for dyadic relationship strain. Because PADLS include more hands-on assistance, the perception of difficulty may create more feelings of embarrassment or stress and impact the perception of relationship strain. Future intervention work may target perceptions of function to improve the dyadic relationship by using techniques such as open communication about difficulties.

